# A Study on the Analgesic Effects of Two Different Doses of Morphine Added to Intrathecal Levobupivacaine in Cesarean Section: A Randomized Double-Blind Study

**DOI:** 10.7759/cureus.102133

**Published:** 2026-01-23

**Authors:** Syama Sundar Ayya, Aparna Jarathi, Mulam Lakshmi Haritha, Rama Krishna Prasad Chikkala, Sandeep Garre

**Affiliations:** 1 Anesthesiology and Critical Care, All India Institute of Medical Sciences, Bibinagar, IND; 2 Obstetrics and Gynecology, All India Institute of Medical Sciences, Bibinagar, IND

**Keywords:** cesarean section, intrathecal morphine, levobupivacaine, postoperative analgesia, spinal anesthesia

## Abstract

Introduction: Intrathecal morphine is considered the gold standard for post-cesarean analgesia. However, opioid-related adverse effects, especially with larger doses, limit its uniform adoption in clinical practice. We aimed to compare the analgesic efficacy and safety profile of two doses of morphine (50 µg and 100 µg) added to intrathecal hyperbaric levobupivacaine in parturients undergoing elective cesarean section.

Materials and methods: This prospective randomized double-blind study included a total of 76 parturients scheduled for elective cesarean section under spinal anesthesia, who were randomized to receive 10 mg of 0.5% hyperbaric levobupivacaine with either 50 µg (Group A) or 100 µg (Group B) of intrathecal morphine. Other outcomes that were assessed include postoperative pain scores, requirement for additional analgesics, and maternal and fetal adverse events.

Results: Following the exclusion of two patients from Group A, 74 patients were included in the statistical analysis (Group A, n=36; Group B, n=38). Demographic characteristics and perioperative hemodynamics were comparable between the two groups. The median time (minutes) to the first analgesic request was similar in both groups (Group A: 363 (interquartile range (IQR): 207-442) vs. Group B: 365 (IQR: 200-445), P=0.931). However, a significantly higher proportion of patients in Group A required supplemental nonsteroidal anti-inflammatory drugs (NSAIDs) during the assessment period. The incidence of maternal adverse effects, such as pruritus (33.3% vs. 21.1%, P=0.234) and postoperative nausea and vomiting (PONV) (13.8% vs. 14.3%, P=0.462), was comparable between the two groups, and none of the patients developed respiratory depression or hypoxia. Perioperative hemodynamic parameters and neonatal outcomes assessed by Apgar scores were similar between groups.

Conclusion: In this study, both 50 µg and 100 µg doses of intrathecal morphine provided effective and safe postoperative analgesia following elective cesarean section. However, the 100 µg dose demonstrated superior analgesia in terms of a reduced requirement for supplemental NSAIDs, rather than a difference in the time to first analgesic request. As this benefit was achieved without an increase in opioid-related side effects, 100 µg of intrathecal morphine may be the preferred dosage for post-cesarean analgesia.

## Introduction

Adequate analgesia after cesarean delivery is essential to facilitate early mobilization, reduce thromboembolic and pulmonary complications, and promote faster recovery [1]. Adequate postoperative pain management supports comfortable breastfeeding, maternal-infant bonding, and active neonatal care, whereas poorly controlled pain increases physiological stress and the risk of chronic post-cesarean pain [2]. Intravenous morphine has an onset of action within 1-2 minutes and a duration of action of approximately 3-4 hours; however, the onset of action of intrathecal morphine is 30-60 minutes, and the duration of analgesia lasts up to 30 hours [3,4]. Intravenous morphine is not preferred in obstetric surgeries since it crosses the placenta and breast milk, risking neonatal respiratory depression and sedation [5]. Hence, intrathecal morphine is currently considered the gold standard for post-cesarean analgesia [6]. The most recent PROSPECT guideline for elective cesarean section recommends 50 µg-100 µg of intrathecal morphine [7]. Despite guideline recommendations advocating the use of neuraxial morphine for cesarean delivery, consistent adoption in routine clinical practice remains limited [8,9]. This reluctance is mainly due to dose-dependent adverse effects of intrathecal morphine, such as nausea, vomiting, pruritus, and, rarely, delayed respiratory depression. These safety concerns, coupled with the need for extended postoperative monitoring, have added to the reluctance to follow guideline-recommended practices.

Spinal anesthesia is preferred for cesarean delivery because it provides reliable sensory and motor blockade, minimal fetal drug exposure, reduces the risk of aspiration and uterine atony, and allows for the immediate initiation of breastfeeding [10,11]. Larger doses of morphine prolong analgesia but are associated with a higher incidence of opioid-related side effects. Several studies have therefore attempted to identify an optimal intrathecal morphine dose that provides adequate postoperative analgesia while minimizing adverse effects. Notably, Carvalho et al. demonstrated that 50 µg of intrathecal morphine provided analgesia comparable to 100 µg, with a lower incidence of side effects in the Brazilian population [12].

The optimal dose of intrathecal morphine remains uncertain, and the balance between prolonged analgesia and increased side effects with higher doses likely varies between populations. Considering the generally shorter body habitus of Indian women, lower doses of intrathecal morphine may be sufficient to achieve adequate post-cesarean analgesia with fewer adverse effects [13]. This gap between guideline recommendations and real-world practice, together with the paucity of Indian data, underscores the need for studies evaluating lower intrathecal morphine doses in this population.

This study was therefore designed to compare the time to first analgesic request and the quality of postoperative analgesia (assessed by the requirement for rescue analgesics), along with the incidence of maternal side effects, between two doses of intrathecal morphine (50 µg and 100 µg) administered with intrathecal levobupivacaine in parturients undergoing elective cesarean section.

## Materials and methods

This prospective, randomized, double-blind study was conducted at a tertiary care teaching hospital after approval from the Institutional Ethics Committee (AIIMS/BBN/IEC/JULY/2022/223-R), and written informed consent was obtained from all participants. The study was registered with the Clinical Trials Registry of India prior to patient recruitment (registration number: CTRI/2023/03/050622). Adult pregnant women aged 20-35 years with a singleton pregnancy beyond 37 weeks of gestation and scheduled for elective cesarean section under spinal anesthesia were included. Exclusion criteria included emergency cesarean section, ASA physical status classes III-V, known bleeding diathesis, neuromuscular disorders, local infection at the injection site, contraindications to neuraxial anesthesia, body mass index (BMI) greater than 40 kg/m^2^, allergy to study medications, and use of psychiatric drugs or chronic pain medications [14].

Participants were randomly allocated using a computer-generated randomization sequence into two groups: Group A (intrathecal morphine 50 µg) and Group B (intrathecal morphine 100 µg). Both patients and outcome assessors were blinded to group allocation. All patients underwent preanesthetic evaluation and received intravenous metoclopramide 10 mg and pantoprazole 40 mg on the morning of surgery. Patients were kept nil per oral according to standard guidelines: eight hours for heavy meals, six hours for light meals, and two hours for clear liquids.

To maintain blinding, the study drug was prepared by an anesthesiologist not involved in patient management or data collection. Under strict aseptic conditions, 1 mL of morphine (10 mg/mL) was diluted with 19 mL of normal saline to obtain a final concentration of 0.5 mg/mL (500 µg/mL). For Group A (50 µg), 0.5 mL of the 0.5 mg/mL solution was drawn into a 1 mL syringe and further diluted with 0.5 mL of normal saline to achieve a concentration of 0.25 mg/mL (250 µg/mL). From this solution, 0.2 mL (50 µg) was added to 2 mL of 0.5% hyperbaric levobupivacaine. For Group B (100 µg), 1 mL of the 0.5 mg/mL solution was drawn into a 1 mL syringe, and 0.2 mL (100 µg) was added to 2 mL of 0.5% hyperbaric levobupivacaine. The total volume of the intrathecal injectate was kept constant at 2.2 mL in both groups. Spinal anesthesia was performed in the sitting or lateral decubitus position at the L3-L4 or L4-L5 interspace using a 25-gauge or 26-gauge Quincke needle after local infiltration with 2% lidocaine. Following successful injection of the study drug, patients were immediately placed in the supine position, and a wedge was positioned under the right hip to facilitate left uterine displacement. Surgery commenced only after an adequate sensory block was achieved.

Intraoperative hemodynamic parameters were recorded at regular intervals. Hypotension, defined as a decrease in systolic blood pressure of more than 20% from baseline, was treated with incremental doses of intravenous Ringer's lactate solution along with phenylephrine or norepinephrine, at the discretion of the treating anesthesiologist. Bradycardia, defined as a heart rate less than 45 beats per minute, was managed with intravenous atropine (0.6 mg) if necessary. After delivery, oxytocin (5-15 international units) diluted in Ringer's lactate was administered to stimulate uterine contraction. In the event of inadequate analgesia or visceral pain during the intraoperative period, including pain associated with uterine exteriorization, patients were planned to receive intravenous fentanyl in bolus doses of 0.5 µg/kg. After surgery, patients were transferred to the postanesthesia care unit (PACU). Patients were discharged from the PACU after achieving a score of 9 on the modified Aldrete postanesthetic recovery scale [15]. All patients were monitored for analgesic efficacy using the visual analog scale (VAS) and for adverse events for 24 hours after surgery [16].

Heart rate, blood pressure, peripheral oxygen saturation, requirement for supplemental analgesics, need for conversion to general anesthesia, and occurrence of adverse events, including postoperative nausea and vomiting (PONV), pruritus, and respiratory depression (defined as a respiratory rate <10 breaths/min or SpO₂ <92%) were recorded during the first 24 postoperative hours. The number of patients who developed pruritus and PONV and required treatment was recorded. Neonatal Apgar scores were recorded at 1 and 5 minutes after delivery [17]. Prior to surgery, patients were instructed to use the VAS (0-10 cm), with 0 representing no pain and 10 representing the worst pain imaginable [16]. VAS scores were recorded at 4, 8, 12, and 24 hours after surgery. PONV and pruritus requiring treatment were recorded at 3, 6, 12, and 24 hours postoperatively.

During the postoperative period, when pain scores were ≥3 on the VAS, paracetamol 15 mg/kg, up to 1000 mg, was administered intravenously and repeated every 8 hours. Pain was reassessed 30 minutes after intravenous paracetamol administration. Inadequate analgesia was defined as a VAS score >3 or a patient request for additional analgesia. Intravenous diclofenac 75 mg was administered as rescue analgesia, with a maximum permitted dose of 150 mg within 24 hours. Intravenous diclofenac was planned to be repeated after a minimum interval of 6 hours if pain persisted. If a patient requested additional analgesia 30 minutes after intravenous diclofenac administration, a bilateral ultrasound-guided transversus abdominis plane block using 0.25% ropivacaine (20 mL on each side) was planned as further rescue analgesia. PONV was treated with ondansetron 4 mg intravenously. Pruritus was managed with intravenous pheniramine (22.75 mg). Patients with pruritus refractory to this treatment were planned to be treated with an intravenous naloxone infusion at a rate of 0.25-1 µg/kg/h.

The primary outcome was the time to first analgesic request, defined as the time from the intrathecal injection to the first request for supplemental analgesics. Secondary outcomes included postoperative pain intensity measured using the VAS from 0 to 10, the total consumption of rescue analgesics (NSAIDs), and the incidence of maternal side effects such as nausea, vomiting, pruritus, respiratory depression, and hypoxia. Neonatal well-being was assessed using Apgar scores at 1 and 5 minutes [17].

The sample size was calculated based on a previous study, assuming no true difference in the time to first analgesic request between the two groups (mean:16.3 hours in both groups), a pooled standard deviation of 7 hours, a power of 80%, and a two-sided significance level of 5%, a sample size of 34 patients per group was required to demonstrate equivalence within a predefined margin of ±5 hours [18]. To account for an anticipated 10% dropout rate, the sample size was increased to 38 patients per group.

Statistical analysis

Data were entered into a Microsoft Excel (Microsoft Corporation, Redmond, WA, USA) spreadsheet and analyzed using Jamovi statistical software, version 2.6 (The Jamovi Project, Sydney, Australia), on a modified intention-to-treat basis. The Shapiro-Wilk test was used to assess the normality of the data. Continuous variables are expressed as mean (standard deviation) when normally distributed and as median (interquartile range (IQR)) when non-normally distributed. Intergroup comparison of age was performed using independent t-tests. Time to first analgesic request, height, weight, body mass index (BMI), VAS scores, and 1-minute and 5-minute Apgar scores were non-normally distributed and were therefore compared using the Mann-Whitney U test. Categorical variables were compared using the chi-square test or Fisher exact test, as appropriate. p-value <0.05 was considered statistically significant. For repeated comparisons of postoperative VAS scores at multiple time points, Bonferroni correction was applied, and a p-value <0.0125 was considered statistically significant.

## Results

**Figure 1:**
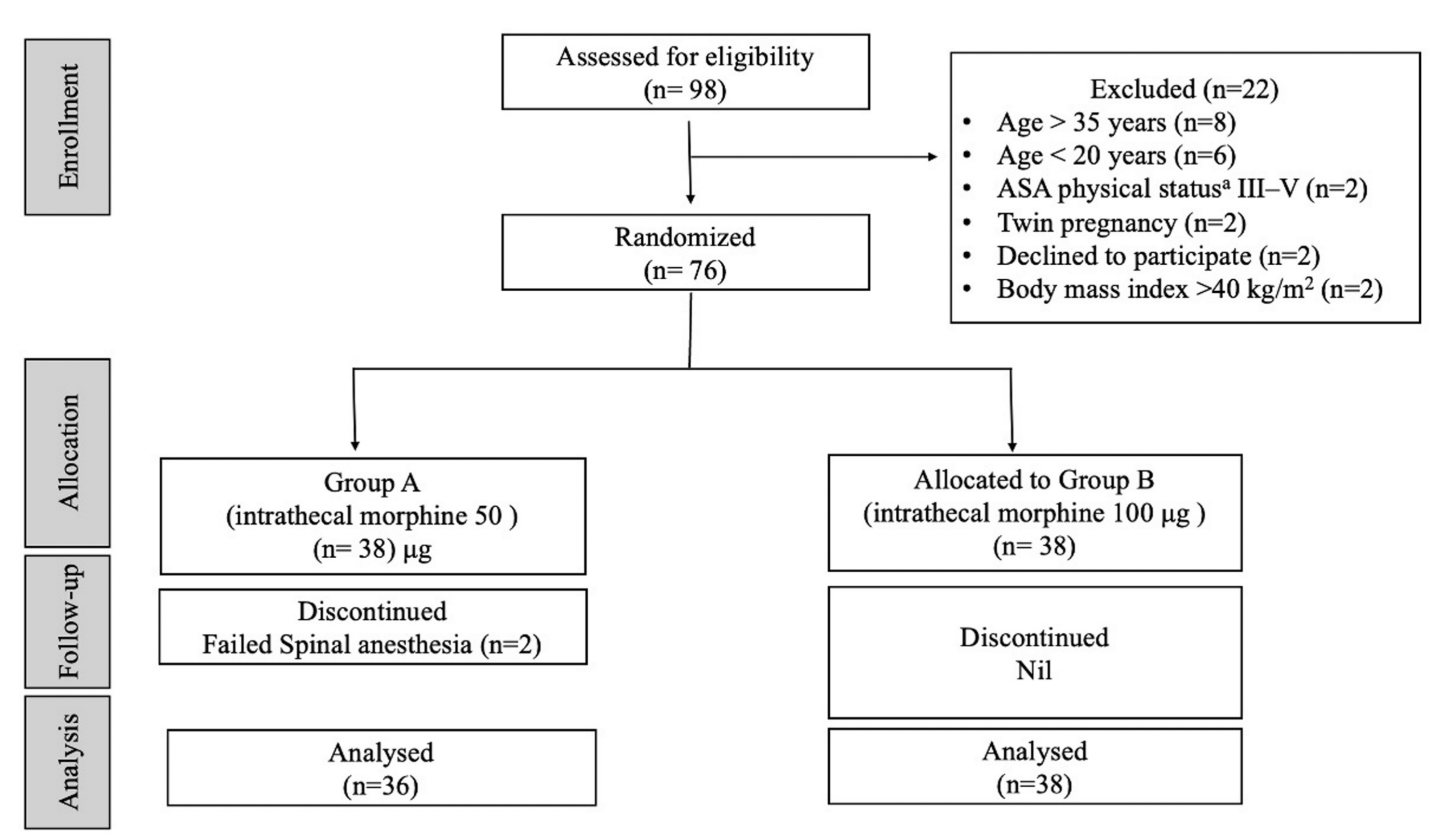
CONSORT flow chart ^a^American Society of Anesthesiologists Physical Status Classification System [11]. CONSORT: consolidated standard of reporting trials

After exclusions, 74 parturients were analyzed, with 36 in Group A (intrathecal morphine 50 µg) and 38 in Group B (intrathecal morphine 100 µg) (Figure 1).

Baseline demographic and anthropometric characteristics were comparable between the two groups (Table 1).

**Table 1:** Characteristics of the study population Data are presented as mean (standard deviation) or median (IQR). Group A included parturients who received intrathecal morphine 50 mcg, and Group B included parturients who received intrathecal morphine 100 mcg. Age was compared between groups using the independent samples t-test, whereas height, weight, and body mass index were compared using the Mann-Whitney U test. BMI: body mass index; IQR: interquartile range

Characteristic	Group A (n=36)	Group B (n=38)	Test statistic	P-value
Age (years)	27.9 (3.51)	27.1 (3.79)	t=0.858	0.394
Height (cm)	155 (151-159)	155 (150-160)	U=679	0.961
Weight (kg)	61.7 (55.8-65.3)	62 (55-67.8)	U=648	0.697
BMI (kg/m^2^)	25.7 (23-28.6)	25.3 (23.1-28.2)	U=679	0.961

The time to first analgesic request did not differ significantly between groups. Postoperative pain scores assessed at 4, 8, 12, and 24 hours were also comparable, with no statistically significant differences at any time point. However, a significantly higher proportion of patients in Group A required supplemental intravenous diclofenac sodium during the first 24 postoperative hours compared with Group B (36.1% vs. 10.5%, P=0.012) (Table 2).

**Table 2:** Comparison of patient outcomes and postoperative complications between groups ^a^Pain was assessed using the VAS [13]. ^b^Apgar score was assessed at 1 and 5 minutes after birth [14]. Data are presented as median (IQR) or number (percentage). Group A included parturients who received intrathecal morphine 50 mcg, and Group B included parturients who received intrathecal morphine 100 mcg. Time to first analgesic request, postoperative VAS scores, and Apgar scores were compared using the Mann-Whitney U test. The incidence of pruritus and PONV was compared using the chi-square test. The number of patients requiring NSAIDs within the first 24 hours after surgery and the incidence of hypotension were compared using Fisher exact test. NSAIDs: non-steroidal anti-inflammatory drugs, PONV: postoperative nausea and vomiting, VAS: visual analogue scale; IQR: interquartile range

Variable	Group A (n=36)	Group B (n=38)	Test statistic	P-value
Time to first analgesic request (minutes)	363 (207-442)	365 (200-445)	U=676	0.931
Postoperative 4^th^ hour VAS^a^	2 (0-3)	2 (0-3)	U=670	0.874
Postoperative 8^th^ hour VAS	2 (2-3)	1(1-3)	U=553	0.149
Postoperative 12^th^ hour VAS	2 (0-3)	2 (1-3)	U=636	0.599
Postoperative 24^th^ hour VAS	2.5 (0.75-3)	2 (1-3)	U=653	0.732
Number of patients required NSAIDs in the first 24 hours after surgery	13 (36.1%)	4 (10.5%)	-	0.012
Pruritus	12 (33.3%)	8 (21.1%)	χ²=1.41	0.234
PONV	5 (13.8%)	5 (14.3%)	χ²=0.540	0.462
Intraoperative hypotension	3 (8.3%)	5 (14.3%)	-	0.712
1-minute Apgar score^b^	8 (8-9)	8 (8-8)	U=612	0.291
5-minute Apgar score	8 (8-9)	8 (8-9)	U=659	0.762

These findings suggest that although the time to first analgesic request was similar between the two doses, intrathecal morphine 100 µg provided better control of breakthrough pain, as reflected by a reduced requirement for supplemental NSAIDs. No patient required a second dose of NSAIDs or a bilateral transversus abdominis plane block during the study period. The incidence of opioid-related adverse effects, including pruritus and PONV, as well as intraoperative hypotension, was similar between groups (Table 2). All episodes of hypotension occurred within 20 minutes of spinal anesthesia administration and were most likely attributable to intrathecal local anesthetic-induced sympathetic blockade. One patient in Group A required a single dose of intravenous pheniramine 22.75 mg for pruritus. One patient required intravenous fentanyl 100 mcg during surgery due to uterine exteriorization; none of the patients received opioids in the postoperative period.

**Figure 2:**
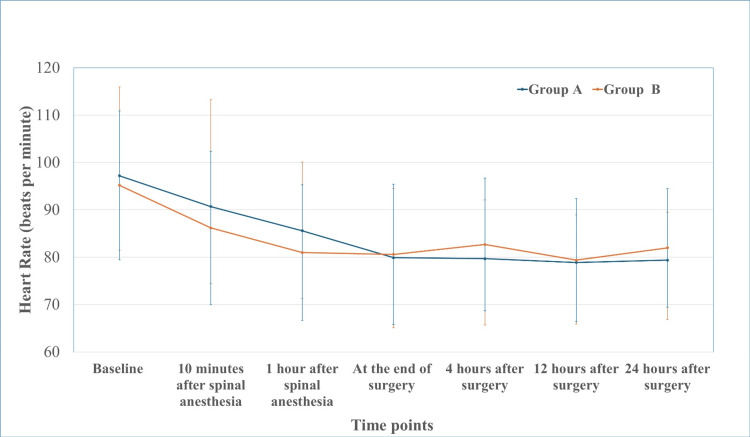
Perioperative heart rate trends in Group A and Group B

Perioperative hemodynamic parameters remained stable and comparable throughout the observation period. Heart rate demonstrated a comparable trend in both groups, without statistically significant differences at any time point (Figure 2).

**Figure 3:**
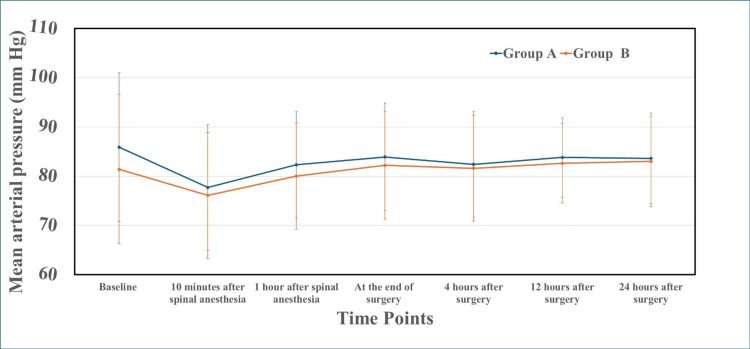
Perioperative mean arterial pressure trends in Group A and Group B

Mean arterial pressure reduced transiently after spinal anesthesia in both groups, then recovered to clinically acceptable levels, with no significant intergroup differences (Figure 3).

**Figure 4:**
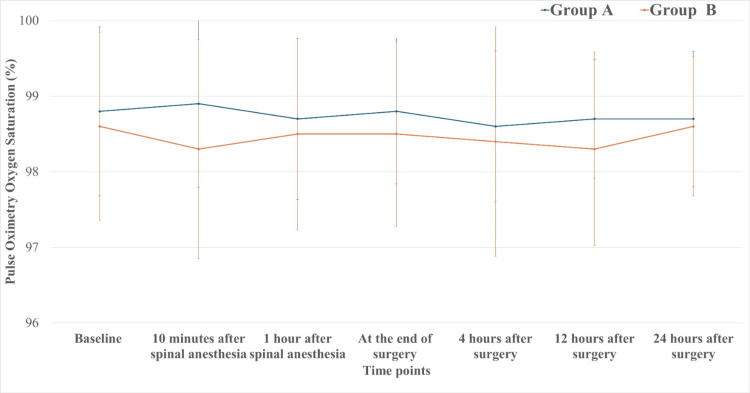
Perioperative pulse oximetry oxygen saturation trends in Group A and Group B

Pulse oximetry oxygen saturation values showed no significant intergroup differences from baseline through 24 hours postoperatively (Figure 4).

No patient developed respiratory depression or hypoxia during the study period. Neonatal outcomes were comparable between groups, with no significant differences in 1-minute or 5-minute Apgar scores (Table 2).

## Discussion

This randomized, double-blind study compared two doses of intrathecal morphine (50 µg and 100 µg) added to levobupivacaine for spinal anesthesia in elective cesarean section. The principal finding of the study is that both doses provided comparable postoperative analgesia, with the time to first analgesic request approximately six hours in both groups. However, the number of patients requiring supplemental NSAIDs was significantly higher in Group A (50 µg) compared to Group B (100 µg). This suggests that while 50 µg is sufficient to delay the initial onset of pain, the 100 µg dose provides an effective block that suppresses breakthrough pain once the local anesthetic effect recedes.

Some previous studies have reported a longer duration to the first analgesic request than observed in our study [18-21]. This discrepancy is likely attributable to the use of multimodal analgesia regimens in those studies, including scheduled paracetamol or patient-controlled opioids initiated intraoperatively, which may have prolonged the time to first analgesic requirement. In contrast, our study design focused strictly on the analgesic efficacy of the intrathecal mixture alone, without the confounding influence of perioperative supplemental analgesics. This approach provides a better understanding of the standalone duration of action for these specific doses of intrathecal morphine.

The optimal dose of intrathecal morphine for cesarean section is often a subject of debate, with clinicians seeking the minimum effective dose that provides adequate analgesia with minimal maternal and fetal adverse effects [20-22]. As noted in a recent editorial, neuraxial morphine continues to be the gold standard for post-cesarean analgesia, utilized in over 80% of hospitals in the United States of America [23]. The article also highlights that historically, higher doses (exceeding 250 µg) were associated with significant risks of respiratory depression, and now, modern obstetric anesthesia has shifted toward identifying the lowest effective dose. Our study contributes to this ongoing refinement by demonstrating that within the "low dose" spectrum (50-100 µg), the dose-response relationship is not strictly linear regarding duration but is significant regarding the quality of postoperative analgesia.

The fear of dose-dependent adverse effects, particularly pruritus, nausea, and respiratory depression, is more imminent with the use of larger doses of intrathecal morphine [24]. Historically, higher doses (>200 µg) were strongly linked to these complications [19,23]. Our study observed no significant difference in the incidence of adverse effects between the 50 µg and 100 µg groups. The incidence of pruritus was slightly higher in the 50 µg group (33.3%) than in the 100 µg group (21.1%), though this difference was not statistically significant (P=0.234). Similarly, PONV rates were nearly identical (~14%). These findings suggest that a dose of 100 µg of intrathecal morphine remains within an acceptable safety margin in this population and may provide superior analgesia without a significant increase in opioid-related adverse effects. It is also worth noting that no patient in either group developed respiratory depression or required high-dependency care, reinforcing the safety profile of these low-dose regimens. The use of levobupivacaine, with its favorable safety profile, combined with these modest morphine doses, resulted in excellent hemodynamic stability across both groups.

An important strength of the present study is its contribution to the limited Indian literature on intrathecal morphine dosing. Although international studies have demonstrated effective analgesia with doses ranging from 50 to 100 µg, extrapolating these findings to the Indian population may not be appropriate due to differences in anthropometry, pain perception, and sociocultural context. Our findings suggest that, within the Indian population, 100 µg of intrathecal morphine provides more sustained analgesia than 50 µg without an increase in opioid-related adverse effects. These results support the need for population-specific dose optimization rather than the uniform adoption of dosing recommendations derived from other populations.

Considering the dose-dependent side-effect profile of neuraxial morphine, a patient-tailored approach is advisable rather than a uniform standard dosing regimen [19,23,25]. High-dose regimens may be preferred for high-risk patients who require deep analgesia and can be closely observed. Conversely, low-dose regimens are likely more appropriate for low-risk patients or in clinical settings where intensive postoperative monitoring is not feasible, ensuring a balance between adequate pain relief and patient safety. The optimal dose of intrathecal morphine may also be influenced by patient-specific factors such as ethnicity, anthropometric characteristics, pain perception, and pharmacogenetic variability. Future studies are needed to identify the impact of these factors on intrathecal morphine dose requirements.

There are limitations to this study. First, the sample size was calculated based on the time to first analgesic request; therefore, the study may not have been powered to detect small statistical differences in less frequent adverse effects. Second, pain assessment was limited to the first 24 hours; evaluating outcomes up to 48 hours might have revealed differences in late-onset side effects or extended analgesic benefits. Finally, this was a single-center study, and findings may vary in populations with different anthropometric characteristics or pain thresholds.

## Conclusions

The time to first analgesic request was comparable between the 50 µg and 100 µg doses of intrathecal morphine; however, the 100 µg dose demonstrated superior control of breakthrough pain, as reflected by a reduced requirement for supplemental NSAIDs. Therefore, 100 µg of intrathecal morphine added to hyperbaric levobupivacaine appears to provide better analgesic efficacy for women undergoing elective caesarean section in the Indian population.
